# Successful treatment with low-dose decitabine in acute myelogenous leukemia in elderly patients over 80 years old: Five case reports

**DOI:** 10.3892/ol.2013.1139

**Published:** 2013-01-18

**Authors:** JIE LIN, HONGLI ZHU, SUXIA LI, HUI FAN, XUECHUN LU, CHENG CHANG, BO GUO, BING ZHAI

**Affiliations:** Department of Geriatric Hematology, Chinese People’s Liberation Army General Hospital, Haidian, Beijing 100853, P.R. China

**Keywords:** decitabine, acute myelogenous leukemia, methylation, elderly

## Abstract

The incidence of acute myelogenous leukemia (AML) in patients over 80 years old is >20 times greater than that observed in younger patients. Previously, no standard treatment protocol for elderly patients with AML existed, however the development of hypomethylating agents, including decitabine, has brought about promising results in AML. In the present study, we report on the usage of a lower than routine dosage of decitabine in patients over 80 years old with AML. Since January 2010, 5 patients diagnosed with AML over the age of 80 years old received treatment with decitabine in our hospital. Decitabine was administered at a dose of 10–15 mg/m^2^ and repeated every other day for a total of 5 days. This cycle was repeated for ∼6 weeks. The 5 patients received a total of 19 cycles of treatment with decitabine. No patient achieved complete or partial remission. An antileukemic effect was observed in 25% of courses (3/12). An increase in platelet count of >20×10^9^/l was observed in 26.3% (5/19) of cycles compared with previous treatment. An increase in hemoglobin concentration of >20 g/l was observed in 36.8% (7/19) of cycles in comparison to previous treatment, four of which achieved normal hemoglobin levels. One patient became red blood cell transfusion-independent. The median survival time was 19.8±4.8 months. Survival time from decitabine administration to mortality was 13.2±5.1 months. The main side-effect was bone marrow suppression with grade III–IV thrombocytopenia, grade III–IV leukocytopenia, grade III–IV neutropenia and anemia accounting for 94.7% (18/19), 47.4% (9/19), 89.5% (17/19) and 21.1% (4/19), respectively. Severe infection or bleeding was not observed and no patient stopped treatment due to adverse effects. In conclusion, extremely low-dose decitabine may be used safely in elderly patients and achieved longer survival times than reported previously in AML patients aged 80 and above. It is suggested that complete remission may not be the primary objective, while improvement of quality of life may be a better choice in AML patients over 80 years old. The cases observed in our study were limited, so more cases are required for further study.

## Introduction

The incidence of acute myelogenous leukemia (AML) in the elderly population is increasing due to the aging of the general population. Statistics from U.S. National Cancer Institute demonstrated that the incidence of AML is 23/100,000 in the age range of 80–84 years, while 21.2/100,000 over the age of 85, which is ∼20 times greater than the incidence in younger patients (1). The biological characteristics, clinical manifestations and treatment response of AML in elderly individuals differs from that in young patients. The major characteristics of AML in elderly patients include: i) high incidence of cytogenetic abnormalities (2–5); ii) high incidence of therapy-related leukemia; iii) association with a prior hematological disease (6); iv) often accompanied by multidrug resistance gene expression (3); v) high incidence of comorbidity and poor tolerance to chemotherapy (7). All of these features contribute to low remission and short survival times. There are no standard treatment regimens for AML in elderly patients. Intensive chemotherapy is not suitable for the treatment of AML in patients over 80 years old (8). Common regimens such as low-dose cytarabine chemotherapy and the best supportive care alone do not prolong survival times significantly in elderly patients. The mean survival time of patients with AML over 80 years old is ∼3 months (9). Therefore, the treatment regimens for elderly patients with AML has evolved from chemotherapy only to targeted therapies. The new drugs under investigation include: humanized anti-CD33 monoclonal antibodies (10), tyrosine kinase inhibitors (11), 5-azacytidine (12), 5-aza-2′-deoxycytidine (13,14), proteasome inhibitors (15), anti-angiogenic drugs (16), FLT3 inhibitors (17) and anti-apoptotic inhibitors (18). Among them, decitabine is a demethylating agent.

Decitabine is also known as 5-aza-2′-deoxycytidine, which is a 2′-deoxycytidine analog. Previous studies have demonstrated that decitabine induces cytotoxicity at high concentrations and demethylation at low concentrations. Decitabine normalizes the demethylation status of cancer suppressor genes by inhibition of DNA methyltransferase-1 (DNMT1), as well as normalizing terminal differentiation, the aging process or apoptosis of cells. In 1992, Zagonel *et al* reported that low-dose decitabine was effective in myelodysplastic syndrome (MDS) and this finding was confirmed in further clinical trials (19,20). Subsequently, the indications for decitabine have been expanded from MDS to AML, chronic myelogenous leukemia and chronic myelomonocytic leukemia (21,22). In previous studies, different degrees of hypermethylation have been identified in leukemia, which are correlated with the development of drug resistance (23). Therefore, demethylation has become the treatment target of leukemia. Currently, most clinical trials for decitabine include only a few patients aged 80 years and above. In addition, the tolerance, efficacy and side-effect profile of decitabine in the Chinese elderly population with AML is unclear due to ethnic differences. The dosage, efficacy and safety of decitabine in this population requires more research. This study observes the short-term efficacy and side-effect profile of decitabine in patients with AML over 80 years old.

## Patients and methods

### Patients

Five elderly patients aged over 80 years old with AML were enrolled in January 2010 and treated with decitabine in the Department of Geriatric Hematology in the People’s Liberation Army General Hospital (Beijing, China). The study was approved by the Ethics Committee of The Chinese PLA General Hospital, Beijing, China. Written informed consent was obtained from the patients priort to the study.

### Treatment

Decitabine (Janssen Pharmaceuticals, Xi’an, China) was administered at a dose of 10–15 mg/m^2^ by continuous intravenous infusion over 1 h and repeated every other day for a total of 5 days. Each cycle was repeated every 6 weeks. No other chemotherapy was provided from administration of decitabine to mortality. Hematopoietic colony-stimulating factors were used during bone marrow suppression. Supportive care measures included the use of red blood cell transfusion in patients with a hemoglobin level <8 g/dl and platelet transfusion in patients with a platelet count <20×10^9^/l or where active bleeding existed.

### Efficacy and side-effect assessment

Blood routine, liver and kidney function tests, chest X-ray, echocardiography, cardiac enzymes and electrocardiograms were performed prior to and following treatment. Bone marrow examination was performed following some cycles of treatment.

Treatment efficacy was evaluated according to the following: i) complete remission (CR): blast cell levels in the bone marrow of <5%, platelet levels of >100×10^9^/l, white blood cell (WBC) levels of >1.5×10^9^/l and absence of extramedullary infiltration. ii) Partial remission (PR): either blast cell levels in the bone marrow between 5–25%, platelet levels of >100×10^9^/l and WBC levels of >1.5×10^9^/l or blast cell levels in the bone marrow of <5% but platelet levels of <100×10^9^/L and WBC levels of <1.5×10^9^/l. iii) Antileukemic effect (ALE): blast cells in the bone marrow reduced by >25% compared with before treatment, but do not meet PR criteria. iv) Progressive disease (PD): blast cells in the peripheral blood or bone marrow increase >25% compared with before treatment. v) Stable disease (SD): other than CR, PR, ALE and PD.

The side-effects of chemotherapy were graded according to WHO criteria: grade 0, grade I, grade II, grade III and grade IV.

### Statistical analysis

Numerical data are reported as mean ± standard deviation and were analyzed with the Student’s t-test (pair-wise comparison for pre- and post-treatment comparison). Event rate was analyzed using the Chi-square test. P<0.05 was considered to indicate a statistically significant result.

## Results

### General data

The clinical information of the 5 patients is presented in Table I. All patients were in relapse or refractory status. Five patients received 19 cycles of treatment with decitabine, mean of which was 4 cycles (range, 3–5 cycles).

### Therapeutic efficacy

Bone marrow examination was performed following 12 cycles of treatment. No patients achieved CR or PR. ALE was observed in 25% of courses (3/12). An increase in platelet count of >20×10^9^/l was observed in 26.3% (5/19) of cycles compared with previous treatment. The mean time to achieve platelet best response was 35.6 days. An increase in hemoglobin concentration of >20 g/l was observed in 36.8% (7/19) of cycles in comparison to previous treatment, four of which achieved normal hemoglobin levels. The mean time to best hemoglobin response was 35.4 days. One patient became red blood cell transfusion-independent.

Median survival time was 19.8±4.8 months (Table I), which was considerably longer than previous studies in elderly AML patients treated with decitabine (24–27) (Table II). The survival time from decitabine administration to mortality was 13.2±5.1 months (Table I).

### Adverse effects

The main side-effect was bone marrow suppression. The lowest value and days to the lowest value of blood routine tests are presented in Table III. Grade III–IV thrombocytopenia, grade III–IV leukocytopenia, grade III–IV neutropenia and anemia accounted for 94.7% (18/19), 47.4% (9/19), 89.5% (17/19) and 21.1% (4/19) of side-effects, respectively. Severe infection or bleeding was not observed. No other grade III–IV side-effects were observed. No patient discontinued treatment due to side-effects.

## Discussion

Abnormalities in DNA methylation play an important role in the development of hematological malignancy. Decitabine, a hypomethalyting agent, reverses hypermethylation of genes *in vitro*. Reversal of aberrant methylation leads to re-expression of silenced tumor suppressor genes. In 2006, the US FDA approved decitabine for the indication of MDS. The efficacy of low-dose decitabine in the treatment for MDS laid the foundation for AML treatment research. Decitabine to treat AML has been evaluated in a number of clinical trials, however these only included a few patients aged 80 years and above.

Elderly patients with AML cannot tolerate either hematopoietic stem cell transplantation or standard intensity chemotherapy. Therefore, regimens with a lesser degree of side-effects are recommended. Furthermore, there is no standard treatment regimen for elderly AML patients. Treatment should be individualized based on the nature of the disease, the physiological status of the patient and the patient’s preferences. We consider that CR may not be the primary objective, while improvement of life quality may be a better choice (9). According to this therapeutic goal, we modified the routine regimen of decitabine. Firstly, the single dose of decitabine was reduced. The total dose equaled 1/2–3/4 of the dose used in routine regimens. Secondly, the administration of decitabine was changed to q.o.d. in order for the adverse effects to be observed sufficiently. Reinforcement of supportive care is also a critical part of treatment. Our study demonstrated that decitabine increases thrombocyte and hemoglobin levels and reduces blast cell levels to some extent in some patients, although none of the patients achieved PR or CR. In particular, the mean survival of the 5 patients was 19.8 months, which is significantly longer than the survival reported for AML patients aged 80 and above (28,29). It is suggested that, although the present decitabine dosage cannot suppress blast cell to normal levels, it may slow down the growth of blast cells. Therefore, although patients do not achieve remission over a long period, disease progression is not rapid.

The main adverse effect of decitabine in elderly patients was bone marrow suppression, most notably in thrombocytopenia and neutrocytopenia. This often occurred 2 weeks following treatment. No severe bleeding or infection was observed following reinforcement of supportive care. If strong supportive care is provided, administration of decitabine in elderly patients is relatively safe.

The cases observed in our study were limited, hence a comprehensive conclusion cannot be made. More cases are required for further study to confirm the results.

## Figures and Tables

**Table I:** Clinical information of patients.

No.	Gender	Age (years)	Classification	Charlson comorbidity index	Chromosome karyotypes	Duration of prior MDS (months)	Survival^a^ (months)	Survival^b^ (months)
1	Male	87	AML-M6	3	46XY	15	14	16
2	Male	86	AML-M2	2	46XY	-	8	27
3	Male	85	AML-M4	2	46XY	30	19	21
4	Male	80	AML-M4	1	45X,-Y	-	8	15
5	Female	82	AML-M4	3	46XX		17	20

a Survival from decitabine administration to mortality;

b Survival from diagnosis to mortality.

-, no history of MDS. AML, acute myelogenous leukemia; MDS, myelodysplastic syndrome.

**Table II:** Regimen of decitabine in elderly AML patients and efficacy.

Authors (Ref.)	Cases	Mean age (years)	Dosage	Response rate (%)	Survival (months)
Cashen, *et al*(24)	55	74	20 mg/m^2^ × 5 days	25	7.7
Blum, *et al*(25)	53	74	20 mg/m^2^ × 10 days	61	13.0
Lübbert, *et al*(26)	227	72	15 mg/m^2^ q8 h × 3 days ATRA 45 mg/m^2^ × 28 days	26	5.5
Garcia-Manero, *et al*(27)	54	60	15 mg/m^2^ × 10 days Valprolic acid	22	6.0

AML, acute myelogenous leukemia; ATRA, all trans-retinoic acid.

**Table III:** Blood routine changes following decitabine treatment (mean ± SD).

Component	Lowest value after treatment	Days to the lowest value after treatment
Platelet	19.6±15.6 (×10^9^/l)	12.9±9.0
White blood cell	2.7±2.5 (×10^9^/l)	14.1±7.9
Absolute neutrophil count	0.5±0.5 (×10^9^/l)	17.7±10.0
Hemoglobin	84.9±7.6 (g/l)	12.4±10.2
